# Asian Lineage of Peste des Petits Ruminants Virus, Africa

**DOI:** 10.3201/eid1707.101216

**Published:** 2011-07

**Authors:** Olivier Kwiatek, Yahia Hassan Ali, Intisar Kamil Saeed, Abdelmelik Ibrahim Khalafalla, Osama Ishag Mohamed, Ali Abu Obeida, Magdi Badawi Abdelrahman, Halima Mohamed Osman, Khalid Mohamed Taha, Zakia Abbas, Mehdi El Harrak, Youssef Lhor, Adama Diallo, Renaud Lancelot, Emmanuel Albina, Geneviève Libeau

**Affiliations:** Author affiliations: Control of Exotic and Emerging Animal Diseases, Montpellier, France (O. Kwiatek, R. Lancelot, E. Albina, G. Libeau);; Central Veterinary Research Laboratories, Soba, Sudan (Y.H. Ali, I.K. Saeed, M.B. Abdelrahman, H.M. Osman, Z. Abbas);; University of Khartoum, Shambat, Sudan (A.I. Khalafalla, A.A. Obeida);; Rabak Veterinary Research Laboratory, White Nile State, Sudan (O.I. Mohamed);; Atbara Veterinary Research Laboratory, River Nile State, Sudan (K.M. Taha);; Biopharama, Rabat, Morocco (M. El Harrak, Y. Lhor);; International Atomic Energy Agency, Vienna, Austria (A. Diallo)

**Keywords:** Peste des petits ruminants virus, phylogeny, epidemiology, sheep, camel, Sudan, Morocco, Africa, viruses, research

## Abstract

Interest in peste des petits ruminants virus (PPRV) has been stimulated by recent changes in its host and geographic distribution. For this study, biological specimens were collected from camels, sheep, and goats clinically suspected of having PPRV infection in Sudan during 2000–2009 and from sheep soon after the first reported outbreaks in Morocco in 2008. Reverse transcription PCR analysis confirmed the wide distribution of PPRV throughout Sudan and spread of the virus in Morocco. Molecular typing of 32 samples positive for PPRV provided strong evidence of the introduction and broad spread of Asian lineage IV. This lineage was defined further by 2 subclusters; one consisted of camel and goat isolates and some of the sheep isolates, while the other contained only sheep isolates, a finding with suggests a genetic bias according to the host. This study provides evidence of the recent spread of PPRV lineage IV in Africa.

*Peste des petits ruminants virus* (PPRV) belongs to the genus *Morbillivirus*, in the family *Paramyxoviridae*. Like other members of the same genus, such as rinderpest virus, *Canine distemper virus*, *Measles virus*, and marine mammal viruses, PPRV is highly pathogenic for its natural hosts (*1*). As consistently reported by the World Organisation for Animal Health, PPRV causes high death rates in livestock. It has a major economic effect, particularly in the intertropical regions of Africa, on the Arabian Peninsula and in the Middle East and Asia (*2*–*5*). The main signs of the acute form of the disease are high fever, mouth ulceration, diarrhea, and pneumonia.

Although rinderpest virus was eradicated after intensive vaccination campaigns in the last quarter of the 20th century (*6*), PPRV has continued to spread in Africa and Asia. In East Africa, outbreaks occurred in 2007 in Uganda and Kenya and in 2009 in Tanzania (*7*); in North Africa, an outbreak occurred in 2008 in Morocco. Genotypic classification of PPRV has identified 4 lineages and appears to be an efficient tool to survey virus spread worldwide. Genetic variability is based on partial sequencing of the fusion (F) protein gene (*8*,*9*) and of the 3′ end of the nucleoprotein (N) gene (*10*). Both are well conserved genes with ≈10% nt mean variability between the most distantly related sequences (*11*); this variability can exceed 30% on some parts of the sequence. However, because the N gene is the most abundantly transcribed virus gene, sensitivity is better achieved with N mRNAs (*12*). Viruses of lineage I and II are restricted to western and central Africa; lineage III is common to eastern Africa and the southern part of the Middle East. In Asia, only viruses of lineage IV have been detected.

In Sudan, continuous outbreaks of PPRV have occurred for >30 years, mainly in sheep and goats (*2*,*13*). Although PPRV infection is well documented in small ruminants, data are rare for other species, such as cattle, buffalo, and camels. In 1997, a PPRV was isolated from pathologic samples collected during a rinderpest-like disease outbreak among buffalo in India (*14*). In September 2004, outbreaks of PPRV in Sudan affected both sheep and camels (*15*,*16*). In camels, a respiratory syndrome was the prominent disease characteristic observed, resembling a previous case reported in Ethiopia during 1995–1996 (*17*).

More recently, in summer 2008, Morocco reported outbreaks of PPRV for the first time (*18*). Because PPRV needs close contact for transmission, this new epizootic was likely the result of introduction of the virus into North Africa through the movement of live infected animals. To characterize PPRV strains identified in Sudan during 2000–2009 and in Morocco during the extensive 2008 outbreaks, a phylogenetic analysis was carried out on wild-type PPRV sequences obtained from biological samples collected from sheep, goats, and camels in these 2 countries.

## Materials and Methods

### Biological Materials

Biological specimens were collected in different parts of Sudan during 2000–2009 from camels, sheep, and goats that showed clinical signs of PPRV. Additional camel specimens were collected after outbreaks associated with high death rates were reported from the Kassala Region in 2004. A total of 80 field samples, including lung, liver, and spleen, were obtained from 49 camels, 26 sheep, and 5 goats with PPRV-like clinical signs. For most of the animals, virus detection and identification were performed directly on the tissue samples collected; however, virus isolation in tissue culture was attempted for some samples. A historical PPRV strain isolated in Sudan in 1971, the Gedarif PPRV strain, was included in this study.

In Morocco, a total of 36 samples were collected from sheep displaying PPRV signs during the 2008 outbreaks. These included oral and ocular swabs and mesenteric lymph nodes, spleen, lung, and whole blood samples.

### Laboratory Investigations

Sample aliquots first were screened for PPRV antigen detection by immunocapture ELISA (ICE test; Biological Diagnostics Supplies, Ltd., Dreghorn, Scotland). A second aliquot was used for viral RNA extraction and virus isolation in tissue culture. Reverse transcription PCR (RT-PCR) amplified the N protein gene directly from tissue samples by using a set of pan-morbillivirus primers as described by Kwiatek et al. (*10*).

For the sequencing of the N gene, the 3′ 351 nt were obtained by employing primers NP3 and NP4 (*19*) and through a modification of the initial protocol by using a 1-step method (OneStep RT-PCR mix; QIAGEN, Courtaboeuf, France). For the F gene, 11 samples were selected from samples that previously tested positive by N amplification. For the specific and sensitive detection of PPRV from these clinical field samples, the initial method developed by Forsyth et al. in 1995 (*8*) was modified by using an additional pair of primer F1/FPPRrev in combination with F1/F2 in a nested PCR. Sequences positions of these primers were 5′-ATCACAGTGTTAAAGCCTGTAGAGG-3′ (PPRV-F1: 777 ± 801), 5′-GAGACTGAGTTTGTGACCTACAAGC-3′ (PPRV-F2: 1124 ± 1148) and 5′-ATATTAATGTGACAAGCCCTAGGGA-3′ (FPPRrev: 2055 ± 2079). The final amplicon was obtained at the expected length of 372 nt after 35 cycles. We analyzed 10 μL of the amplified products by electrophoresis on 1.5% agarose gel. For all positive results, 40 μL of the final product was used directly for sequencing (GATC Biotech, Constance, Germany). All sequences were deposited in GenBank under accession nos. HQ131917– HQ131958. FPPRrev and the study of the target sites of primers F1 and F2 (*8*) were designed by a comparative sequence analysis of various morbillivirus F sequences from the full genomes available in GenBank (accession nos. X74443, EU267273, EU267274, AY560591, FJ905304, and AJ849636).

### Sequence Data, Alignment, and Phylogenetic Analysis

Sequencing reactions were performed by GATC Biotech. Each nucleic acid segment on the N and F genes of the Sudan and Morocco strains was aligned with other sequences from PPRV maintained in the database or retrieved from GenBank (Table 1) (*10*,*20*–*22*). Multiple alignments of 255 and 322 nt of the N and F genes, respectively, were made by using the ClustalW program (www.ebi.ac.uk/clustalw). Phylogenetic analysis was carried out by using the criterion of neighborhood based on the principle of parsimony (*23*). Dissimilarities and distances between the sequences first were determined with Darwin software (*24*). Tree construction was based on the unweighted neighbor-joining method proposed by Gascuel (*25*). Trees were generated with the TreeConMATRIXW program of Darwin (*26*). Bootstrap confidence intervals were calculated on 1,000 replicates.

**Table 1:** Peste des petits ruminant virus (genus *Morbillivirus*) strains and sequences retrieved from GenBank, Africa, 2000–2009*

Lineage	Origin	Year of isolation	Source	GenBank accession no.	
N gene	F gene
I	Senegal	1968	ISRA/Senegal	DQ840165	NA
III	Sudan	1972	CVRL/Sudan	DQ840158	NA
II	Nigeria	1975	IAH/UK	DQ840161	NA
II	Nigeria	1975	IAH/UK	DQ840162	NA
II	Nigeria	1975	IAH/UK; CIRAD/France	DQ840160	X74443
II	Nigeria	1976	IAH/UK	DQ840163	NA
II	Nigeria	1976	IAH/UK	DQ840164	EU267274
II	Ghana	1978	IAH/UK	DQ840167	NA
II	Ghana	1978	IAH/UK	DQ840166	NA
III	Oman	1983	IAH/UK	DQ840168	NA
III	United Arab Emirates	1986	AAZA/UAE	DQ840169	NA
I	Burkina Faso	1988	CIRAD/France	DQ840172	NA
I	Guinea	1988	CIRAD/France	DQ840170	NA
I	Côte d'Ivoire	1989	CIRAD/France	DQ840199	EU267273
I	Guinea-Bissau	1989	CIRAD/France	DQ840171	NA
IV	Israel	1993	KVI/Israel	DQ840173	NA
I	Senegal	1994	ISRA/Senegal	DQ840174	NA
III	Ethiopia	1994	CIRAD/France	DQ840175	NA
IV	India	1994	NPRI/India	DQ840176	NA
IV	India	1994	CIRAD/France	DQ840179	NA
IV	India	1994	CIRAD/France	DQ840180	NA
IV	India	1995	CIRAD/France	DQ840177	NA
IV	India	1995	CIRAD/France	DQ840178	NA
IV	India	1995	CIRAD/France	DQ840182	NA
IV	Israel	1995	KVI/Israel	DQ840181	NA
III	Ethiopia	1996	CIRAD/France	DQ840183	NA
IV	Turkey	1996	CIRAD/France	DQ840184	NA
IV	India	1996	IVRI/India	AY560591	GQ452015
IV	Cameroon	1997	LANAVET/Cameroon	HQ131960	NA
IV	Iran	1998	CIRAD/France	DQ840185	NA
IV	Iran	1998	CIRAD/France	DQ840186	NA
IV	Israel	1998	KVI/Israel	DQ840191	NA
IV	Israel	1998	KVI/Israel	DQ840188	NA
IV	Israel	1998	KVI/Israel	DQ840189	NA
IV	Israel	1998	KVI/Israel	DQ840190	NA
II	Mali	1999	LCV/Mali	DQ840192	NA
IV	Saudi Arabia	1999	CIRAD/France	DQ840195	NA
IV	Saudi Arabia	1999	CIRAD/France	DQ840197	NA
IV	Tajikistan	2004	CIRAD/France	DQ840198	NA
IV	Central African Republic	2004	CIRAD/France	HQ131962	NA
IV	India	2005	CVSH/India	DQ267188	DQ267183
IV	India	2005	CVSH/India	DQ267191	DQ267186
IV	India	2005	CVSH/India	DQ267192	DQ267187
IV	India	2005	CVSH/India	DQ267189	DQ267184
IV	India	2005	CVSH/India	DQ267190	DQ267185
IV	China	2007	NEADDC/China	EU068731	EU816772
IV	China	2007	NEADDC/China	EU340363	EU815053
IV	Bangladesh	2009	DPBAU/Bangladesh	HQ131961	NA
II	Senegal	2010	ISRA/Senegal	HQ131963	NA
0	Kenya	2010	IAH/UK	Z30697	NA

*ISRA, Institut Sénégalais de Recherches Agricoles; NA, GenBank accession no. not available; CVRL, Central Veterinary Research Laboratory; IAH, Institute For Animal Health; CIRAD, Centre de Coopération Internationale en Recherche Agronomique pour le Développement; AAZA, Al Ain Zoo and Aquarium, Al Ain/Abu Dhabi, United Arab Emirates; KVI, Kimron Veterinary Institute; NPRE, National Project on Rinderpest Eradication; IVRI, Indian Veterinary Research Institute; LANAVET, Laboratoire National Veterinaire; LCV, Laboratoire Central Vétérinaire; CVSH, College of Veterinary Science and Husbandry; NEADDC, National Exotic Disease Diagnosis Center; DPBAU, Department of Pathology Bangladesh Agricultural University.

### Virus Isolation

For the samples from Sudan, isolation was successful on MDBK cells with lung samples from Cam_8, Cam_169, Cam_318, Ov_Soba, and Ov_Al Azaza, collected from 3 camels and 2 sheep, respectively. For the samples from Morocco, PPRV Morocco_08_02 virus was isolated successfully from a sheep lung sample after infection of Vero.DogSLAMtag cells (*27*). These isolates were sequenced and compared as described above.

## Results

### Detection of PPRV by N gene RT-PCR

A total of 80 animals from Sudan that initially tested positive in the ICE test were analyzed further by using RT-PCR. Results for 64 animals were positive, including 21 sheep (80.8%), 5 goats (100%), and 38 camels (77.6%) drawn from all of the regions studied: Khartoum, Blue Nile, Northern Sudan, Kassala, Kordofan, and Darfur (Table 2). Of the 36 samples tested from Morocco, 16 yielded a positive RT-PCR.

**Table 2:** Number of field samples with positive results by reverse transcription PCR for peste des petits ruminant virus, by animal and province, among 80 animals sampled in Sudan, 2000–2009*

Region	Sheep			Goats			Camels	
	No. tested	No. (%) positive		No. tested	No. (%) positive		No. tested	No. (%) positive
Khartoum	5	4 (80)		1	1 (100)		NS	NS
Blue Nile	10	7 (70)		3	3 (100)		18	13 (72.2)
Northern Sudan	4	4 (100)		1	1 (100)		21	16 (76.2)
Kassala	5	5 (100)		NS	NS		9	8 (88.9)
Kordofan	1	1 (100)		NS	NS		NS	NS
Darfur	1	0		NS	NS		1	1 (100)
Total	26	21 (80.8)		5	5 (100)		49	38 (77.6)

*Proportion of animals positive for peste des petits ruminant virus in sampling sites according to Sudan regions: Khartoum: 5/6; Blue Nile: Al Azaza, 1/1; Al Hilalia, 1/1; Gezira /Bashagra, 1/1; Tambool, 13/18; White Nile (Sennar/Rabak), 7/10; Northern: River Nile, Atbara, 16/21; Ed Damar, 4/4; Dongola, 1/1; Kassala: Gedarif, 3/4, Kassala, 7/7, Abudelaiq, 3/3. Darfur: Alfashir 0/1, Nyala, 1/1; Kordofan: El Nihood, 1/1. NS, species not sampled in region.

### Characterization of Strains Involved in the Infection of Sheep, Goats, and Camels in Sudan, and in Sheep in Morocco

Of the tissue samples tested, lung and lymph nodes were the most suitable for sequencing because they had the highest viral load, thereby yielding a sufficient amount of PCR product. Sequence also was obtained from several virus isolates, including the historical Gedarif isolate from 1971. The partial N gene sequences were obtained for 26 of the 64 samples from Sudan and 6 of the 16 samples from Morocco. Sequences were aligned with an extended set of PPRV isolate sequences that either were in the database or were retrieved from GenBank where they were described by Kwiatek et al. (*10*) and Banyard et al. (*20*).

**Figure 1:**
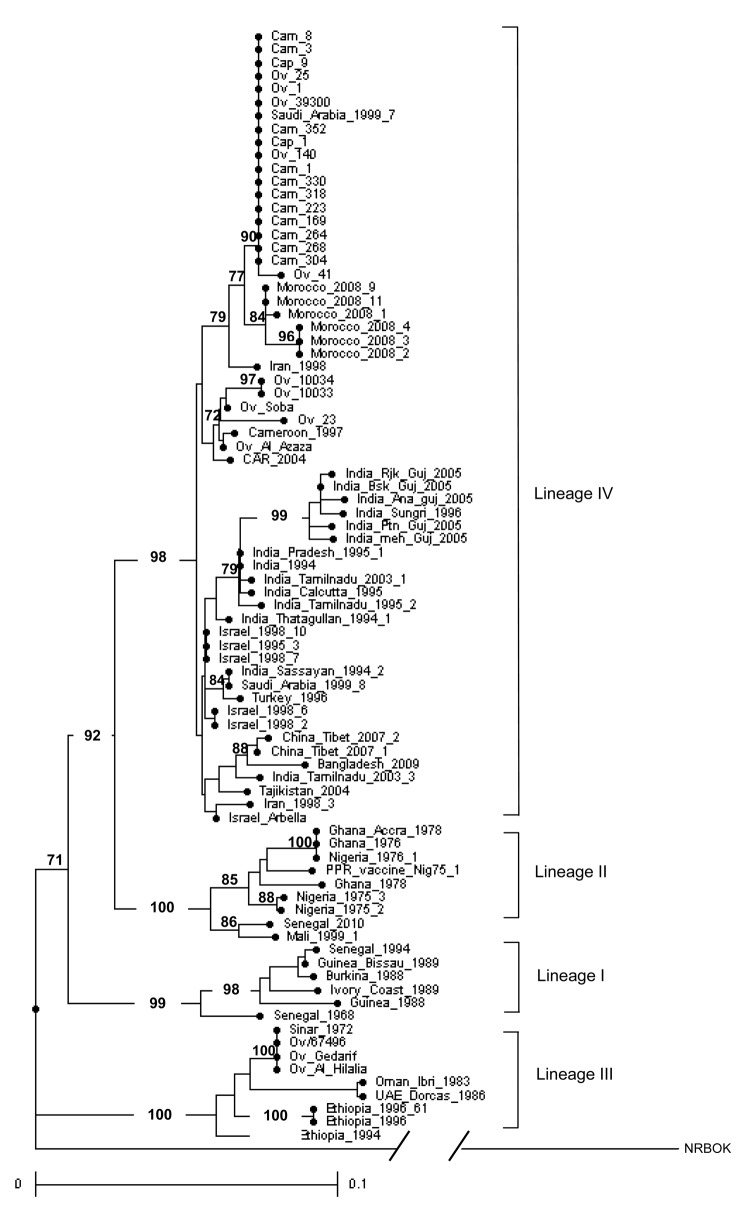
Phylogenetic analysis of the 1232–1560 nt sequence of the N protein gene of sequenced peste des petits ruminant (PPR) virus strains. The phylogram was generated by analyzing 1,000 bootstrap replicates; clusters were supported by bootstrap values >70%. Strains from Sudan are represented by prefixes: cam, camel; cap, caprine; ov, ovine. The Kabete 0 strain of rinderpest (RBOK) vaccine strain of rinderpest virus retrieved in GenBank (accession no. Z30697) was used as an outgroup. Scale bar indicates nucleotide substitutions per site.

In contrast to what would have been expected for isolates from eastern Africa, most of the PPRV strains collected in Sudan during 2000–2009 were clustered in lineage IV (24); only a few remained in lineage III (2). Molecular typing also showed for the first time the presence of PPRV lineage IV in Morocco (Figure 1).

Lineage IV isolates from Sudan could be further distinguished into 2 clusters. The grouping of the sequences within the clusters was supported by bootstrap percentages >70%. In the first group, 17 sequences from all camel and goat isolates and some sheep isolates (camel Sudan Cam_1, Cam_3, Cam_8, Cam_169, Cam_223, Cam_264, Cam_268, Cam_304, Cam_330, Cam_352, Cam_318; sheep Ov_1, Ov_140, Ov_25, Ov_39300; goat Cap_9, Cap_1) showed 100% identity and matched with Saudi Arabia_1999_7 strain. Sheep Sudan Ov_41 differed from the previous sequences by 2 nt at position 68 (C to T) and 142 (A to G). The divergence of this cluster with consensus IV ranged from 1.2% (Cam_318) to 2% (Ov_41). Furthermore, the Saudi Arabia cluster was related closely to the 6 strains from Morocco collected from the 2008 outbreak, the closest differing by 4 nt.

A separate cluster containing only sheep samples collected during 2000–2008 (sheep Sudan Ov_10033, Ov_10034, Ov_23, Ov_Soba, Ov_Al Azaza) matched with 2 strains from central Africa (Cameroon_1997 [CAMER_1997] and Central African Republic_2004 [CAR_2004]) (*20*). This cluster was less homogeneous than the cluster previously described; nucleotide variations from the lineage IV consensus sequence ranged from 1.6% (Ov_Al Azaza) to 3.9% (Ov_23).

Only 2 isolates collected at the start of the study (in mid-2000) from sheep in western and eastern Sudan fell into lineage III. Sequences of these 2 field isolates remained similar to the historical strains of Sinnar 72 that was isolated 40 years earlier from the Blue Nile region (*2*), and with the Gedarif PPRV strain that was isolated from sheep in 1971 (*13*). In addition, these isolate sequences were close to an Omani strain, Ibri_1983 (*28*) and a United Arab Emirates strain, Dorcas_1986 (*29*), but they differed from these strains with a nucleotide variation of 4.7%.

**Figure 2:**
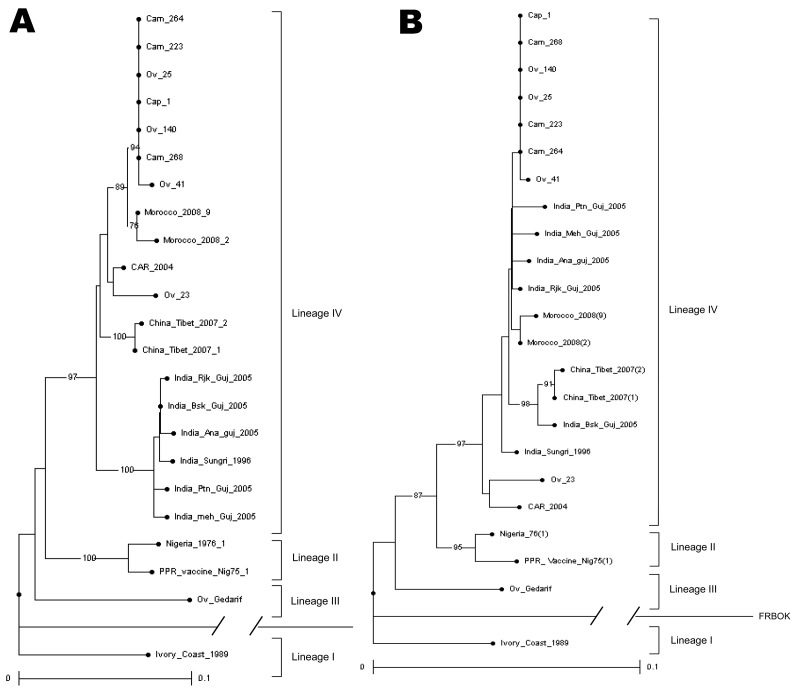
Phylogenetic analysis of the 3′ end nucleotide sequence of the N protein gene (A) and of the 777–1148 nt sequence of the F protein gene (B) of 11 peste des petits ruminant (PPR) virus samples selected from 2 lineage IV clusters and from lineage III as defined in Figure 1. Other designated strains were as published (*10*,*20*–*22*). The phylogram was generated by analyzing 1,000 bootstrap replicates; clusters were supported by bootstrap percentages >70%. Strains from Sudan are represented by prefixes: cam, camel; cap, caprine; ov, ovine. The Kabete 0 strain of rinderpest (RBOK) vaccine strain of rinderpest virus retrieved in GenBank (accession no. Z30697) was used as an outgroup. Scale bars indicate nucleotide substitutions per site.

Because the dataset within the Saudi Arabia cluster was highly homogeneous, we compared an N phylogenetic tree (Figure 2, panel A) with an F phylogenetic tree (Figure 2, panel B) on a panel of viruses. These viruses were selected from the 2 lineage IV clusters defined in Figure 1 and from lineage III with additional PPRV from Asia, for which both gene sequences were available (*21*,*22*). The F phylogenetic tree confirmed the high homology within the Saudi Arabia cluster, and the cluster’s closeness to strains from Morocco. It also allowed for assignment of strains from Sudan to the 2 described clusters represented by the Arabia_1999_7 and the Central Africa strains.

Unlike the N gene amplifications, amplications with F-specific primers (*8*) resulted in a number of negative samples by RT-PCR because of mismatches occurring mainly in the F2 reverse primer (Table 3). To circumvent this problem, another reverse primer (FPPRrev) was designed that matched with all lineages.

**Table 3:** Multiple sequence alignment of F1/F2 primers and FPPRrev target sites from different PPRV isolates of lineage II compared with isolates of lineage IV*

PPRV isolate†	F1 (primer 5′), nt 777–801	F2 (primer 3′), nt 1124–1148	FPPRrev (primer 3′), nt 2055–2079
NIGERIA 75_1‡	ATCACAGTGTTAAAGCCTGTAGAGG	GCTTGTAGGTCACAAACTCAGTCTC	TCCCTAGGGCTTGTCACATTAATAT
NIGERIA 76_1	-------------------------	---G---------------------	-------------------------
ICV 89	-------------------------	-------------------------	-------------------------
SUNGRI 96	-------------------------	---G-----------G---G-C---	-------------------------
China/TibetGeg07-30	--------------A----------	---G-----C-----G---G-C---	-------------------------
TURKEY 00	-------------------------	---G-----------G---G-C---	-------------------------

*FPPRrev, new reverse primer designed in this study; PPRV, peste des petits ruminants virus. Nucleotide position (numbered according to GenBank accession no. X74443 sequence). The consensus sequence corresponds to the F gene of the PPRV Nigeria 75_1 vaccine strain. F1/F2 primers from (*8*). †Lineage and GenBank accession nos.: NIGERIA 75_1, lineage II, X74443; NIGERIA 76_1, lineage II, EU267274; ICV 89, lineage I, EU267273; SUNGRI 96, lineage IV, AY560591, China/TibetGeg07-30, lineage IV, FJ905304; TURKEY 00, lineage IV, AJ849636. ‡Vaccine strain.

**Figure 3:**
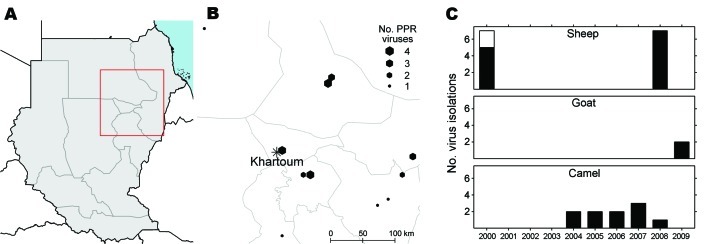
Distribution of samples positive for peste des petits ruminant (PPR) virus by reverse transcription PCR in Sudan for which lineage identification could be done, Sudan. A) Location of samples (red box) in Sudan. B) Locations and numbers of positive samples. C) Time distribution of virus isolations, by animal species.

The sequences and the corresponding lineage classification generated in this study were collated to field information. Occurrence among species of the different lineages show that most of the isolates belong to lineage IV; all camel isolates grouped in the cluster Saudi Arabia, whereas only sheep isolates were found within the other cluster, Central Africa. Only sheep isolates were found in lineage III (Table 4). Geographic and times distribution according to animal species and lineage in Sudan during 2000–2009 show that lineage IV was circulating as early as mid-2000 and that progressive substitution of lineage III in a large zone encompassing the eastern, northern, Blue Nile, and Khartoum regions has taken place since this date (Figure 3).

**Table 4:** PPRV sequences analyzed from tissue samples of sheep, goats, and camels in Sudan and from sheep in Morocco, with lineage classifications, 1971 and 2000–2009*

Sample no.	Sequence available	Coordinates		Lineage classification	GenBank accession nos.		Source	Year
		X	Y		N gene	F gene		
Ov_39300	PPRV/Kuku/Khartoum/ KHSUD00-1	15.617	32.6	IV-SA	HQ131933	–	Lung	2000
Cam_1	PPRV/Kassala/KSUD04-1	15.45	36.4	IV-SA	HQ131935	–	Lung	2004
Cam_3	PPRV/Kassala/KSUD04-2	15.45	36.4	IV-SA	HQ131947	–	Lung	2004
Cam_223	PPRV/Atbara/NSUD05-1	17.701	33.99	IV-SA	HQ131934	HQ131949	Lung	2005
Cam_268	PPRV/Atbara/NSUD05-2	17.701	33.99	IV-SA	HQ131936	HQ131951	Lung	2005
Cam_264	PPRV/Atbara/NSUD05-3	17.701	33.99	IV-SA	HQ131948	HQ131950	Lung	2005
Cam_304	PPRV/Tambool/BNSUD06-1	14.933	33.4	IV-SA	HQ131937	–	Lung	2006
Cam_330	PPRV/Tambool/BNUD06-2	14.933	33.4	IV-SA	HQ131938	–	Lung	2006
Cam_8	PPRV/Kassala/KSUD07	15.45	36.4	IV-SA	HQ131939	–	Isolate	2007
Cam_169	PPRV/Tambool/BNSUD07-1	14.933	33.4	IV-SA	HQ131940	–	Isolate	2007
Cam_318	PPRV/Tambool/BNSUD0-2	14.933	33.4	IV-SA	HQ131941	–	Isolate	2007
Cam_352	PPRV/Atbara/NSUD08	17.701	33.99	IV-SA	HQ131942	–	Lung	2008
Ov_1	PPRV/Abudelaiq/KSUD08	14.967	35.92	IV-SA	HQ131922	–	Lung	2008
Ov_25	PPRV/Bashagra/Gezira/ BNSUD08	14.912	33.24	IV-SA	HQ131943	HQ131955	Lung	2008
Ov_140	PPRV/Gedarif/KSUD08	14.033	35.38	IV-SA	HQ131944	HQ131953	Lung	2008
Cap_1	PPRV/Rabak/BNSUD09	13.18	32.74	IV-SA	HQ131945	HQ131954	Lung/liver	2009
Cap_9	PPRV/Dongola/NSUD09	19.169	30.47	IV-SA	HQ131932	NS	Lung	2009
Ov_41	PPRV/Ed Damar/NSUD08	17.593	33.96	IV-SA + 2 mut	HQ131931	HQ131959	Lung	2008
Ov_10033	PPRV/Ed Damar/NSUD00-1	17.593	33.96	IV-SA	HQ131929	NS	Lung	2000
Ov_10034	PPRV/Ed Damar/NSUD00-2	17.593	33.96	IV-SA	HQ131930	NS	Lung	2000
Ov_Soba	PPRV/Soba/Khartoum/ KHSUD00-2	15.51	32.63	IV-SA	HQ131920	NS	Isolate	2000
Ov_ Al Azaza	PPRV/Al Azaza/BNSUD00	14.204	35.54	IV-SA	HQ131917	NS	Isolate	2000
Ov_23	PPRV/Soba/Khartoum/ KHSUD08	15.51	32.63	IV-SA	HQ131921	HQ131952	Spleen	2008
Ov_Gedarif	PPRV/Gedarif/KSUD71	14.033	35.38	III	HQ131918	HQ131956	Isolate	1971
Ov_Al Hilalia	PPRV/Al Hilalia/BNSUD00	14.921	33.23	III	HQ131919	NS	Isolate	2000
Ov_67496	PPRV/Abudelaiq/KSUD00	14.967	35.92	III	HQ131946	NS	Isolate	2000
Morocco_2008_1	PPRV/Morocco08-01	33.56	–6.89	IV	HQ131923	NS	Lymph	2008
Morocco_2008_2	PPRV/Morocco08-02	33.56	–6.89	IV	HQ131924	HQ131957	Lung	2008
Morocco_2008_3	PPRV/Morocco08-03	33.56	–6.89	IV	HQ131925	NS	Lymph	2008
Morocco_2008_4	PPRV/Morocco08-04	33.56	–6.89	IV	HQ131926	NS	Lymph	2008
Morocco_2008_9	PPRV/Morocco08-09	34.03	–6.8	IV	HQ131927	HQ131958	Lung	2008
Morocco_2008_11	PPRV/Morocco08-11	30.4	–9.6	IV	HQ131928	NS	Lung	2008

*PPRV, peste des petits ruminants virus; ov, ovine; IV-SA, lineage IV Saudi Arabia cluster; lung, lung tissue sample; KHSUD, Khartoum Region Sudan; cam, camel; KSUD, Kassala Sudan; NSUD, Northern Sudan; BNSUD, Blue Nile Sudan; isolate, virus isolate; cap, caprine; liver, liver tissue sample; NS, not sequenced; IV-SA + 2 mut, lineage IV, differing from Saudi Arabia cluster by 2 mutations; IV-SA, lineage IV Central Africa cluster; spleen, spleen tissue sample; lymph, lymph node sample.

## Discussion

This study presents findings from PPRV surveillance in Sudan over a 10-year period and the results of tests conducted in Sudan and in Morocco during Morocco’s 2008 outbreaks (*18*). Analysis of 80 samples confirmed the wide distribution of PPRV throughout Sudan, which has been known since 1971 (*2*,*13*,*15*). Genetic characterization of 26 samples positive for PPRV also provided strong evidence of the introduction and spread of Asia PPRV lineage IV in the country. Surprisingly, the number of samples containing the indigenous lineage III decreased dramatically during the study.

Viruses collected in Morocco also were classified as lineage IV. The origin of the outbreaks in Morocco remains unknown, although Ayari-Fakhfakh et al. (*30*) recently reported a PPRV seroprevalence in Tunisia of up to 7.45%, indicating that the virus is present across a wider area of northern Africa.

Analysis of the N and F genes showed that 2 clusters were identified in strains from Sudan: 1 related to a strain from Saudi Arabia, the other related to central Africa viruses. The 6 strains from Morocco collected during the 2008 outbreaks were related closely to the Saudi Arabia cluster.

Within the Saudi Arabia cluster, a remarkable genetic stability over a decade was observed; notably, all camel isolates fell into this cluster. The variability and nucleotide sequence was slightly higher for isolates in the central Africa cluster, within which only sheep were found. The inconsistent low genetic variability of isolates from Sudan may be a consequence of a species bias because of the limited contact between camels and sheep. When replicated in a single host that has a limited exposure to new variants, the viral genome thus may remain highly conservative. This hypothesis was verified previously for the measles virus over a shorter period (1997–2000). For the measles virus, it appears that the lower the level of circulation, the higher the sequence conservation (*31*).

Camels were not regarded as possible hosts for PPRV until 1992, when a number of authors reported PPRV seroconversion in these animals (*32*–*36*). The first documented outbreak of PPRV in camels, reported from Ethiopia in 1996, consisted of highly contagious respiratory syndromes with high illness rates but low death rates (*17*,*37*). The causative agent was confirmed to be a lineage III PPRV (A. Diallo, unpub. data). This present study confirmed the etiology of the disease in camels through the virologic and epidemiologic investigations and the isolation of PPRV. Surveillance of camels furthermore allowed the virus to be detected in consecutive outbreaks in Kassala, eastern Sudan (2004); Atbara, northern Sudan (2005); and Tambool, Blue Nile region, Sudan (2007) (*16*). Viruses from the same cluster also were recovered from sick sheep and goats in a large zone encompassing the eastern, northern, Blue Nile, and Khartoum regions as early as mid-2000. Under extensive pastoral farming conditions in these regions, camels may have served as a bridge with areas of northern Africa and contributed to the spread of a camel-derived strain of lineage IV, as seen in Morocco. In contrast, none of the camel and goat isolates were distributed in the central Africa cluster, although viruses of this cluster were cocirculating with camel viruses during the same period and in the same areas of northern Sudan, Khartoum, and Blue Nile.

The reason that isolates of lineage IV became predominant in Sudan, progressively replacing lineage III viruses, during the last decade remains unclear. Lineage IV has been present in Asia and in part of the Middle East for a long time, probably as low virulent strains (*5*). However, a constant rise of disease incidence recently has been associated with this lineage and suggests increased virulence (*3*). A virulent lineage IV strain may have been introduced in Africa during the 1990s, resulting in outbreaks in both camels and small ruminants. In parallel, rinderpest virus control and eradication may have favored the decline of cross-immunity in small ruminants and their increased risk for PPRV as predicted earlier by Taylor (*38*).

## References

[1] Gibbs EPJ, Taylor WP, Lawman MPJ, Bryant J. Classification of the peste des petits ruminants virus as the fourth member of the genus *Morbillivirus.* Intervirology. 1979;11:268–74. 10.1159/000149044 457363

[2] El Hag Ali B, Taylor WP. Isolation of peste des petits ruminants virus from Sudan. Res Vet Sci. 1984;36:1–4. 6200906

[3] Shaila MS, Shamaki D, Forsyth MA, Diallo A, Kitching RP, Barrett T. Geographic distribution and epidemiology of peste des petits ruminants virus. Virus Res. 1996;43:149–53. 10.1016/0168-1702(96)01312-3 8864204

[4] Lefèvre PC, Diallo A. Peste des petits ruminants. Rev Sci Tech. 1990;9:935–81. 2132714 10.20506/rst.9.4.532

[5] Taylor WP, Barrett T. Peste de petits ruminants and rinderpest. In: Aitken ID, editor. Diseases of sheep, 4th ed. Oxford: Blackwells Science; 2007. p. 460–8.

[6] Roeder PL. The animal story. BMJ. 2005;331:1262–4. 10.1136/bmj.331.7527.1262 16308392 PMC1289332

[7] World Organisation for Animal Health. Immediate notifications and follow-up reports. World Animal Health Information Database (WAHID) Interface; 2009 [cited 2009 Jan 27]. http://www.oie.int/wahis/public.php?page=reports_pdf_download

[8] Forsyth MA, Barrett T. Evaluation of polymerase chain reaction for the detection and characterisation of rinderpest and peste des petits ruminants viruses for epidemiological studies. Virus Res. 1995;39:151–63. 10.1016/0168-1702(95)00076-3 8837881

[9] Ozkul A, Akca Y, Alkan F, Barrett T, Karaoglu T, Dagalp SB, Prevalence, distribution, and host range of peste des petits ruminants virus, Turkey. Emerg Infect Dis. 2002;8:708–12. 12095439 10.3201/eid0807.010471 PMC2730320

[10] Kwiatek O, Minet C, Grillet C, Hurard C, Carlsson E, Karimov B, Peste des petits ruminants (PPR) outbreak in Tajikistan. J Comp Pathol. 2007;136:111–9. 10.1016/j.jcpa.2006.12.002 17321539

[11] Chard LS, Bailey DS, Dash P, Banyard AC, Barrett T. Full genome sequences of two virulent strains of peste-des-petits ruminants virus, the Côte d'Ivoire 1989 and Nigeria 1976 strains. Virus Res. 2008;136:192–7. 10.1016/j.virusres.2008.04.018 18541325

[12] Barrett T, Banyard AC, Diallo A. Molecular biology of the morbilliviruses. In: Barrett T, Pastouret PP, Taylor WP, editors. Rinderpest and peste des petits ruminants virus. London: Academic Press; 2006. p. 31–56.

[13] Ali B el-H. A natural outbreak of rinderpest involving sheep, goats and cattle in Sudan. [**PMID: 4807688**]. Bull Epizoot Dis Afr. 1973;12:421–8. 4807688

[14] Govindarajan R, Koteeswaran A, Venugopalan AT, Shyam G, Shaouna S, Shaila MS, Isolation of peste des petits ruminants virus from an outbreak in Indian buffalo (*Bubalus bubalis*). Vet Rec. 1997;141:573–4. 10.1136/vr.141.22.573 9423239

[15] Saeed IK, Ali YH, Khalafalla AI, Rahman-Mahasin EA. Current situation of peste des petits ruminants (PPR) in the Sudan. Trop Anim Health Prod. 2010;42:89–93. 10.1007/s11250-009-9389-5 19548103

[16] Khalafalla AI, Saeed IK, Ali YH, Abdurrahman MB, Kwiatek O, Libeau G, An outbreak of peste des petits ruminants (PPR) in camels in the Sudan. Acta Trop. 2010;116:161–5. 10.1016/j.actatropica.2010.08.002 20707980

[17] Roger F, Guebre Yesus M, Libeau G, Diallo A, Yigezu LM, Yilma T. Detection of antibodies of rinderpest and peste des petits ruminants viruses (*Paramyxoviridae, Morbillivirus*) during a new epizootic disease in Ethiopian camels (*Camelus dromedarius*). Rev Med Vet (Toulouse). 2001;152:265–8.

[18] Sanz-Alvarez J, Diallo A, De La Rocque S, Pinto J, Thevenet S, Lubroth J. Peste des petits ruminants (PPR) in Morocco. EMPRES Watch, 2008 August 1–7 [cited 2009 Jan 27]. ftp://ftp.fao.org/docrep/fao/011/aj120f/aj120f00.pdf

[19] Couacy-Hymann E, Roger F, Hurard C, Guillou JP, Libeau G, Diallo A. Rapid and sensitive detection of peste des petits ruminants virus by a polymerase chain reaction assay. J Virol Methods. 2002;100:17–25. 10.1016/S0166-0934(01)00386-X 11742649

[20] Banyard AC, Parida S, Batten C, Oura C, Kwiatek O, Libeau G. Global distribution of peste des petits ruminants virus and prospects for improved diagnosis and control. J Gen Virol. 2010;91:2885–97. 10.1099/vir.0.025841-0 20844089

[21] Kerur N, Jhala MK, Joshi CG. Genetic characterization of Indian peste des petits ruminants virus (PPRV) by sequencing and phylogenetic analysis of fusion protein and nucleoprotein gene segments. Res Vet Sci. 2008;85:176–83. 10.1016/j.rvsc.2007.07.007 17850836

[22] Wang Z, Bao J, Wu X, Liu Y, Li L, Liu C, Peste des petits ruminants virus in Tibet, China. Emerg Infect Dis. 2009;15:299–301. 10.3201/eid1502.080817 19193278 PMC2657621

[23] Saitou N, Nei M. The neighbor-joining method: a new method for reconstructing phylogenetic trees. Mol Biol Evol. 1987;4:406–25. 3447015 10.1093/oxfordjournals.molbev.a040454

[24] Perrier X, Flori A, Bonnet F. Data analysis methods. In: Hamos P, Seguin M, Perrier X, Glaszmann JC, editors. Genetic diversity of cultivated tropical plants. Montpellier (France): Gifield Science Publishers; 2003. p. 43–76.

[25] Gascuel O. BIONJ: an improved version of the NJ algorithm based on a simple model of sequence data. Mol Biol Evol. 1997;14:685–95. 9254330 10.1093/oxfordjournals.molbev.a025808

[26] Van de peer Y, DeWatchter R. Treecon: a software package for the construction and drawing of evolutionary trees. Comput Appl Biosci. 1993;9:177–82. 8481820 10.1093/bioinformatics/9.2.177

[27] Seki F, Ono N, Yamaguchi R, Yanagi Y. Efficient isolation of wild strains of canine distemper virus in Vero cells expressing canine SLAM (CD150) and their adaptability to marmoset B95a cells. J Virol. 2003;77:9943–50. 10.1128/JVI.77.18.9943-9950.2003 12941904 PMC224612

[28] Taylor WP, al Busaidy S, Barrett T. The epidemiology of peste des petits ruminants in the Sultanate of Oman. Vet Microbiol. 1990;22:341–52. 10.1016/0378-1135(90)90021-M 2114052

[29] Furley CW, Taylor WP, Obi TU. An outbreak of peste des petits ruminants in a zoological collection. Vet Rec. 1987;121:443–7. 10.1136/vr.121.19.443 3424615

[30] Ayari-Fakhfakh E, Ghram A, Bouattour A, Larbi I, Gribâa-Dridi L, Kwiatek O, First serological investigation of peste-des-petits ruminants and Rift Valley fever in Tunisia. [**PMID: 20167519**]. Vet J. 2011;187:402–4. 10.1016/j.tvjl.2010.01.007 20167519

[31] El Mubarak HS, van de Bildt MW, Mustafa OA, Vos HW, Mukhtar MM, Ibrahim SA, Genetic characterization of wild-type measles viruses circulating in suburban Khartoum, 1997–2000. J Gen Virol. 2002;83:1437–43. 12029159 10.1099/0022-1317-83-6-1437

[32] Ismail TM, Hassas HB, Nawal M, Rakha GM, Abd El-Halim MM, Fatebia MM. Studies on prevalence of rinderpest and peste des petits ruminants antibodies in camel sera in Egypt. Vet Med J Giza. 1992;10:49–53.

[33] Haroun M, Hajer I, Mukhtar M, Ali BE. Detection of antibodies against peste des petits ruminants virus in sera of cattle, camels, sheep and goats in Sudan. Vet Res Commun. 2002;26:537–41. 10.1023/A:1020239515020 12416868

[34] Abraham G, Sintayehu A, Libeau G, Albina E, Roger F, Laekemariam Y, Antibody seroprevalences against peste des petits ruminants (PPR) virus in camels, cattle, goats, and sheep in Ethiopia. Prev Vet Med. 2005;70:51–7. 10.1016/j.prevetmed.2005.02.011 15967242

[35] Abubakar A, Sanda AB, El-Yuduga A, Baba SS. Seroprevalence of Morbillivirus antibody and abattoir survey of one-humped slaughtered camels (*Camelus dromedarius*) in Maiduguri municipal abattoir, Maiduguri, Nigeria. Asian J Sci Res. 2008;1:85–9. 10.3923/ajsr.2008.85.89

[36] Albayrak H, Gür S. A serologic investigation for peste des petits ruminants infection in sheep, cattle, and camels (*Camelus dromedarius*) in Aydın province, West Anatolia. Trop Anim Health Prod. 2010;42:151–3. 10.1007/s11250-009-9400-1 19554466

[37] Roger F, Yigezu LM, Hurard C, Libeau G, Mebratu G, Diallo A, Investigations on a new pathological condition of camels in Ethiopia. Journal of Camel Practice and Research. 2000;7:163–5.

[38] Taylor WP. The distribution and epidemiology of peste des petits ruminants. Prev Vet Med. 1984;2:157–66. 10.1016/0167-5877(84)90059-X

